# Closing the Gap: Increases in Life Expectancy among Treated HIV-Positive Individuals in the United States and Canada

**DOI:** 10.1371/journal.pone.0081355

**Published:** 2013-12-18

**Authors:** Hasina Samji, Angela Cescon, Robert S. Hogg, Sharada P. Modur, Keri N. Althoff, Kate Buchacz, Ann N. Burchell, Mardge Cohen, Kelly A. Gebo, M. John Gill, Amy Justice, Gregory Kirk, Marina B. Klein, P. Todd Korthuis, Jeff Martin, Sonia Napravnik, Sean B. Rourke, Timothy R. Sterling, Michael J. Silverberg, Stephen Deeks, Lisa P. Jacobson, Ronald J. Bosch, Mari M. Kitahata, James J. Goedert, Richard Moore, Stephen J. Gange, for The North American AIDS Cohort Collaboration on Research and Design (NA-ACCORD) of IeDEA

**Affiliations:** 1 British Columbia Centre for Excellence in HIV/AIDS, Vancouver, British Columbia, Canada; 2 Simon Fraser University, Burnaby, British Columbia, Canada; 3 Johns Hopkins University, Baltimore, Maryland, United States of America; 4 Centers for Disease Control and Prevention, Atlanta, Georgia, United States of America; 5 Ontario HIV Treatment Network, Toronto, Ontario, Canada; 6 The Core Center, Bureau of Health Services of Cook County, Chicago, Illinois, United States of America; 7 University of Calgary, Calgary, Alberta, Canada; 8 Veterans Administration Connecticut Healthcare System and Yale University, West Haven, Connecticut, United States of America; 9 McGill University Health Centre, Montreal, Quebec, Canada; 10 Oregon Health and Science University, Portland, Oregon, United States of America; 11 University of California San Francisco, San Francisco, California, United States of America; 12 University of North Carolina at Chapel Hill, Chapel Hill, North Carolina, United States of America; 13 Vanderbilt University, Nashville, Tennessee, United States of America; 14 Kaiser Permanente Northern California, Oakland, California, United States of America; 15 San Francisco General Hospital, University of California San Francisco, San Francisco, California, United States of America; 16 Harvard School of Public Health, Boston, Massachusetts, United States of America; 17 University of Washington, Seattle, Washington, United States of America; 18 National Cancer Institute, Rockville, Maryland, United States of America; Infectious Disease Service, United States of America

## Abstract

**Background:**

Combination antiretroviral therapy (ART) has significantly increased survival among HIV-positive adults in the United States (U.S.) and Canada, but gains in life expectancy for this region have not been well characterized. We aim to estimate temporal changes in life expectancy among HIV-positive adults on ART from 2000–2007 in the U.S. and Canada.

**Methods:**

Participants were from the North American AIDS Cohort Collaboration on Research and Design (NA-ACCORD), aged ≥20 years and on ART. Mortality rates were calculated using participants' person-time from January 1, 2000 or ART initiation until death, loss to follow-up, or administrative censoring December 31, 2007. Life expectancy at age 20, defined as the average number of additional years that a person of a specific age will live, provided the current age-specific mortality rates remain constant, was estimated using abridged life tables.

**Results:**

The crude mortality rate was 19.8/1,000 person-years, among 22,937 individuals contributing 82,022 person-years and 1,622 deaths. Life expectancy increased from 36.1 [standard error (SE) 0.5] to 51.4 [SE 0.5] years from 2000–2002 to 2006–2007. Men and women had comparable life expectancies in all periods except the last (2006–2007). Life expectancy was lower for individuals with a history of injection drug use, non-whites, and in patients with baseline CD4 counts <350 cells/mm^3^.

**Conclusions:**

A 20-year-old HIV-positive adult on ART in the U.S. or Canada is expected to live into their early 70 s, a life expectancy approaching that of the general population. Differences by sex, race, HIV transmission risk group, and CD4 count remain.

## Introduction

Since the introduction of combination antiretroviral therapy (ART), there have been considerable improvements in survival among HIV-positive individuals, as regimens have become more effective, simpler, and better tolerated [1]–[3]. The health gains associated with ART use have been substantial at both the individual and societal level [1], [2]. ART is effective in increasing the life span of HIV-positive individuals [2] and is associated with a reduction in new infections [4]–[6]. However, in tandem with increases in life expectancy following the introduction of ART, HIV-positive individuals are increasingly experiencing age-related co-morbid conditions, which are impacting both the length and quality of their lives [7], [8]. Studies show a small but persistent gap in the life span between HIV-positive and -negative individuals, particularly within key affected populations [2], [9]–[11].

In the general populations of Canada and the United States (U.S.), 2009 estimates of life expectancy at age 20 years were 59.7 and 57.0 years for men and 63.9 and 61.7 years for women, respectively [12]. While ART has led to significant increases in survival among HIV-positive adults globally, the effect of ART on life expectancy in the U.S. and Canada has not been well characterized. No study has had a sufficient sample size to determine whether gains in life expectancy for HIV-positive individuals are similar to those observed in the general population, or are similar across sex, race, or transmission groups. The objective of this study is to examine temporal changes in life expectancy from 2000 to 2007 among HIV-positive individuals on ART in the U.S. and Canada and to compare life expectancy by selected sociodemographic and clinical characteristics.

## Methods

### Study Population

Estimates of life expectancy were obtained from mortality rates from the North American AIDS Cohort Collaboration on Research and Design (NA-ACCORD), a multi-site collaboration of interval and clinical cohorts of HIV-positive individuals in Canada and the U.S. NA-ACCORD is the North American regional collaboration sponsored by the National Institute of Health's International Epidemiological Databases to Evaluate AIDS (IeDEA) consortium. Details on the NA-ACCORD collaboration and participating cohort studies have been published previously [13]. Briefly, each contributing cohort has developed standardized cohort-specific methods of data collection. At scheduled intervals, these cohorts submit data regarding participants' demographic characteristics, ART prescription information, dates and results of laboratory tests including HIV-1 RNA (viral load) and CD4 count, clinical diagnoses, and vital status. These data are transferred securely to the NA-ACCORD central Data Management Core, where they undergo quality control for completeness and accuracy before they are combined into harmonized data files. Quality control includes instituting measures to reduce the probability that an individual was participating in more than one cohort.

HIV-positive individuals in NA-ACCORD were included in this analysis if they were aged 20 years or older (due to small numbers at younger ages) at the start of each period, had no prior antiretroviral therapy experience when observed to initiate ART, and had a CD4 cell count measurement at or within six months following ART initiation (participating NA-ACCORD sites are described in Appendix S1). ART was defined as a multi-class regimen containing at least three antiretroviral drugs, including a protease inhibitor, a non-nucleoside reverse-transcriptase inhibitor, or three nucleoside reverse-transcriptase inhibitors. The study period was restricted to 1 January 2000 and 31 December 2007, a time period often characterized as the modern ART era [14], in order to produce estimates that are more relevant to the current context of HIV antiretroviral management. All study participants were included either when they were first dispensed ART, or from the start of the study period if initiation was pre-2000, regardless of whether they later discontinued or modified their therapeutic regimen. The human subjects activities of the NA-ACCORD has been approved by the Johns Hopkins School of Medicine institutional review board (NA_00002683) as well as the local institutional review boards at each of the participating cohorts. Additional information on ethical approval and consent procedures for each individual cohort is described in Appendix S1.

### Primary Outcome: Life Expectancy

Life expectancy is defined as the average number of additional years that a person of a specific age will live, if the current age-specific mortality rates remain constant over the course of the individual's lifetime [15]. We interpret life expectancy as a summary measure of age-specific mortality patterns in our defined population [16], [17]. Although single year-of-age life tables are used to calculate life expectancy for large populations such as the entire U.S. population with complete death capture via a vital statistics registry [18], we used abridged life tables (with data aggregated into age intervals) due to the relatively small size of our study population and number of deaths therein [15]. Each participating cohort has procedures in place for the ascertainment of deaths for their participants, although these methods are not uniform across all cohorts. Information about deaths among NA-ACCORD participants was obtained from each participating cohort, either through linkages with vital statistics registries, physician report obtained in medical record, or through active follow-up. A sensitivity analysis estimated mortality rates restricting to cohorts that obtained information on deaths from linkage to vital statistics registries.

### Statistical Analyses

Mortality rates are the building blocks of life expectancy estimates. We calculated all-cause mortality rates (per 1,000 person-years) by dividing the total number of deaths by the total number of person-years of observation. Person-years for estimating mortality rates were accumulated from ART initiation (or January 1, 2000 for those who initiated prior to this date) until death date, loss to follow-up (defined as 6 months after the participant's last CD4 cell count or viral load measurement), or December 31, 2007, whichever came first. Each participant's person-years contribution was partitioned into three calendar eras (1/1/2000–12/31/2002, 1/1/2003–12/31/2005, and 1/1/2006–12/31/2007) and four age groups (20–34, 35–44, 45–54, ≥55 years), categorizations designed to ensure that cells had sufficient number of deaths to calculate rates of death. We further stratified the calendar- and age-specific mortality rates by sex (male vs. female), race (non-white vs. white), HIV transmission risk (men who have sex with men (MSM), injection drug use (IDU) including MSM who inject drugs, and other transmission groups), and baseline CD4 cell count measured at, or within 6 months of, ART initiation (<350 cells/mm^3^ vs. ≥350 cells/mm^3^) to examine differences in life expectancy by these characteristics.

Mortality rates are susceptible to bias from informative censoring. To address this potential source of bias, we used weighted regression methods to estimate mortality rates [19] and compared the life expectancy estimated using unweighted and weighted regression methods for mortality rates, which are described in more detail in Appendix S2. Abridged life tables constructed as part of this study were based on widely used and standardized methods employed by Chiang [15] and outlined in greater detail in an earlier study [2]. Estimates of life expectancy, stratified by several indicators described above, were taken at age 20 years for individuals on ART in the NA-ACCORD. These estimates were calculated under the assumption that any deaths (and therefore person-years contributed from the decedents) occur uniformly in any defined calendar period [15].

## Results

Our study population consisted of 22,937 treatment-naive ART initiators age ≥20 years. Participants contributed 82,022 person-years and 1,622 deaths for an overall unweighted (i.e., without accounting for censoring weights) mortality rate of 19.8 [95% Confidence Interval (CI): 18.8, 20.8] per 1,000 person-years. In a sensitivity analysis restricted to only those who were observed to initiate ART during our study period (18,591 participants contributing 1,057 deaths), the overall unweighted mortality rate was 18.9 (95% CI: 17.8, 20.1) per 1,000 person-years.

**Table 1** describes the demographic characteristics of included participants, overall and by calendar period. As individuals can contribute person-time to a number of periods, these calendar periods are not mutually exclusive. Of the entire sample, 77% were male, 20% had a history of IDU, 62% were non-white, and 72% initiated ART with a CD4 cell count <350 cells/mm^3^. Other than age, demographic and clinical characteristics remained stable across periods.

**Table 1:** Demographic and clinical characteristics of participants, overall and for those contributing to each calendar period (n = 22,937).

	Period 1 (2000–2002) n (%)	Period 2 (2003–2005) n (%)	Period 3 (2006–2007) n (%)	Overall (2000–2007) n (%)
**Age at start of ART**				
20–34	2,774(25)	3,331(21)	3,036(17)	5,808(25)
35–44	4,938 (45)	6,670 (42)	6,679 (38)	9,622 (42)
45–54	2,486 (23)	4,248 (27)	5,737 (32)	5,692 (25)
55+	743 (7)	1,464 (9)	2,307 (13)	1,823 (8)
**Sex**				
Female	2,509 (23)	3,733 (24)	4,206 (24)	5,352 (23)
Male	8,432 (77)	11,980 (76)	13,553 (76)	17,585 (77)
**Mode of transmission**				
Injection drug use	2,470 (23)	3,219 (20)	3,513 (20)	4,684 (20)
MSM	4,324 (40)	5,990 (38)	6,911 (39)	8,842 (39)
Other	4,147 (38)	6,504 (41)	7,335 (41)	9,411 (41)
**Race**				
White	4,410 (40)	6,214 (40)	6,808 (38)	8,643 (38)
Non-white	6,531 (60)	9,499 (60)	10,953 (62)	14,294 (62)
**CD4 cell count at start of ART (cells/mm^3^)**				
<350	7,578 (69)	11,402 (73)	12,884 (73)	16,615 (72)
≥350	3,363 (31)	4,311 (27)	4,875 (28)	6,322 (28)

Note: MSM, *men who have sex with men*; ART, *combination antiretroviral therapy*.

**Table 2** characterizes the study sample in terms of number of deaths and unweighted mortality rates (per 1,000 person-years). In general, mortality rates were highest among individuals with IDU history (34.5, 95% CI: 31.9, 37.4) compared with MSM and other transmission groups, non-whites (22.4, 95% CI: 21.1, 23.8) compared with whites, and individuals with CD4 counts <350 cells/mm^3^ (23.3, 95% CI: 22.1, 24.6) compared with those with CD4 ≥350 cells/mm^3^.

**Table 2:** Population size, deaths, and unweighted mortality rate, overall and by select categories, 2000 to 2007.

	Population	Deaths	Person Years	Unweighted mortality rate*	(95% CI**)
**Overall**	22,937	1,622	82,022	19.8	(18.8, 20.8)
**Sex**					
Female	5,352	366	19,171	19.1	(17.2, 21.2)
Male	17,585	1,256	62,851	20.0	(18.9, 21.1)
**Mode of transmission**					
Injection drug use	4,684	598	17,326	34.5	(31.9, 37.4)
Men who have sex with men	8,842	403	32,139	12.5	(11.4, 13.8)
Other transmission groups	9,411	621	32,508	19.1	(17.7, 20.7)
**Race**					
White	14,294	539	33,717	16.0	(14.7, 17.4)
Non-white	8,643	1,083	48,305	22.4	(21.1, 23.8)
**CD4 cell count at start of ART (cells/mm^3^)**					
<350	16,615	1,351	58,003	23.3	(22.1, 24.6)
≥350	6,322	271	24,019	11.3	(10.0, 12.7)

Per 1,000 person-years.

CI: Confidence Interval.

Note: ART, *combination antiretroviral therapy*.

**Figure 1:**
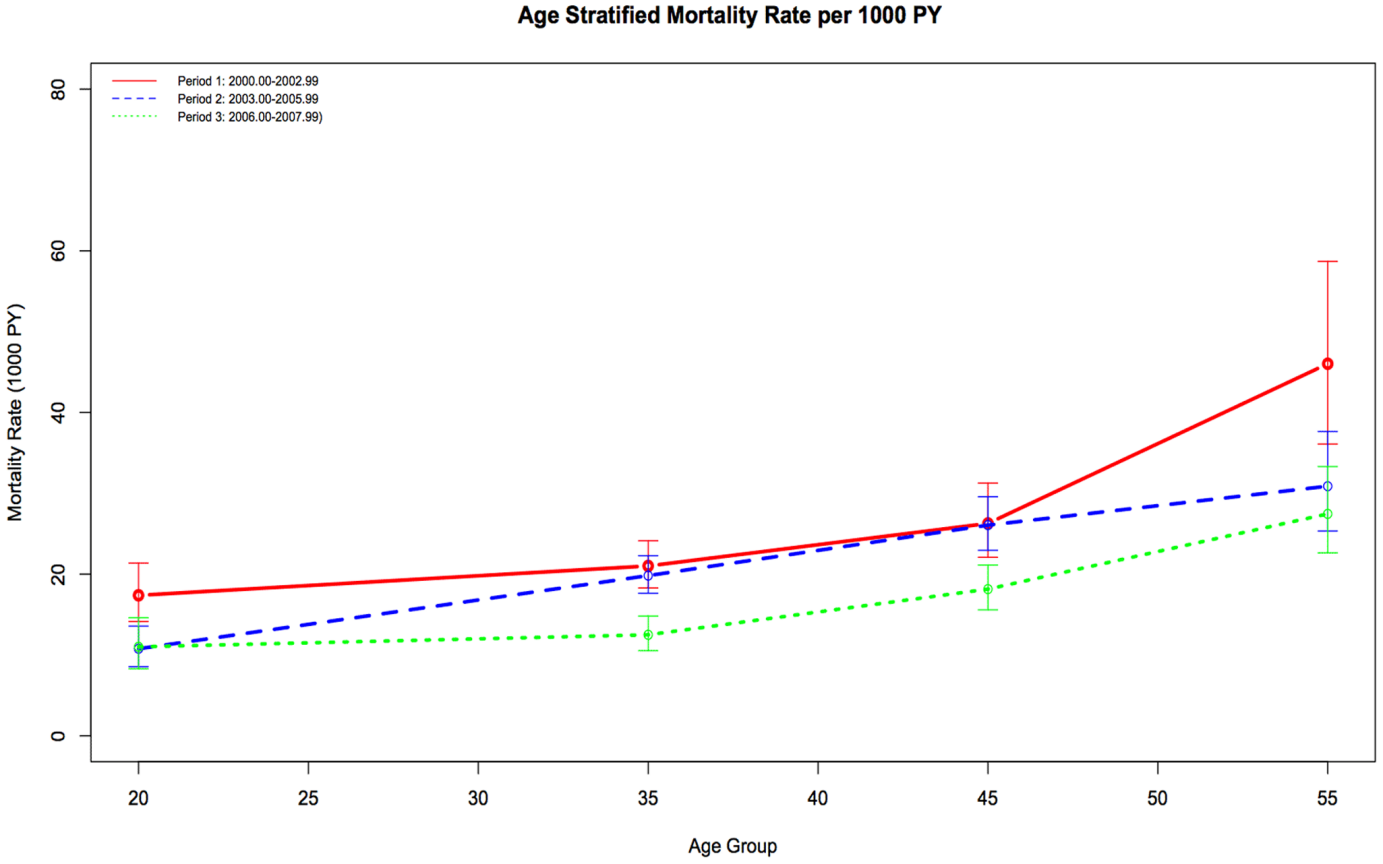
Age-specific unweighted mortality rates (per 1000 person-years), by calendar-period, among individuals on ART in the NA-ACCORD, 2000–2007. Note: PY, person-years; ART, combination antiretroviral therapy.

**Figure 1** shows unweighted age-specific mortality rates for the three periods under observation. As expected, age-specific mortality rates were highest in the earliest period and lowest in the most recent period. In a sensitivity analysis, we estimated mortality rates in fourteen participating cohorts that collect information about deaths by linking to death registries to four that do not; although we see a slightly higher mortality rate among cohorts with death registry matches, the impact on life expectancy estimates appears minimal (data not shown) (see Appendix S3 for more information).

**Table 3** presents overall life expectancy estimates based on unweighted mortality rates and mortality rates weighted for loss to follow-up. The unweighted and weighted life expectancy estimates were similar, with overall life expectancy estimates of 42.6 and 42.7 years, respectively; however the standard error was slightly larger when the unweighted mortality rates were used (SE 0.2 vs. SE 0.1). Over the entire study period, women had similar life expectancies to men, IDUs had lower life expectancies than MSM and individuals with other risk factors, non-whites had lower life expectancies than whites, and those with CD4 count <350 cells/mm^3^ had a lower life expectancy than those with CD4 count ≥350 cells/mm^3^. Finally, in a sensitivity analysis (further described in Appendix S4), when the sample was restricted to those who initiated therapy after 2000, life expectancy at 20 years increased to 45.1 years (SE 0.3).

**Table 3:** Life expectancy estimates (LEE) and standard errors (SE) at age 20 years using weighted and unweighted mortality rates, 2000 to 2007.

	Unweighted (LEE, SE)	Weighted* (LEE, SE)
**Overall**	42.6 (0.2)	42.7 (0.1)
**Sex**		
Female	43.7 (0.5)	43.9 (0.3)
Male	42.4 (0.3)	42.4 (0.2)
**Mode of transmission**		
Injection drug use	29.0 (0.5)	29.1 (0.2)
Men who have sex with men	56.9 (0.5)	57.3 (0.3)
Other transmission groups	49.7 (0.5)	50.1 (0.3)
**Race**		
White	52.0 (0.4)	52.1 (0.2)
Non-white	38.0 (0.3)	38.0 (0.1)
**CD4 cell count at start of ART (cells/mm^3^)**		
<350	38.7 (0.3)	38.8 (0.14)
≥350	54.4 (0.5)	54.6 (0.27)

Note: LEE, *life expectancy estimate (years)*; SE, *standard error*; ART, *combination antiretroviral therapy*.

In effort to reduce bias from informative censoring, we used weighted regression methods to estimate mortality rates, using an indicator variable that was created to identify participants who were lost to follow-up. Further details are available in Appendix S2.

**Figure 2:**
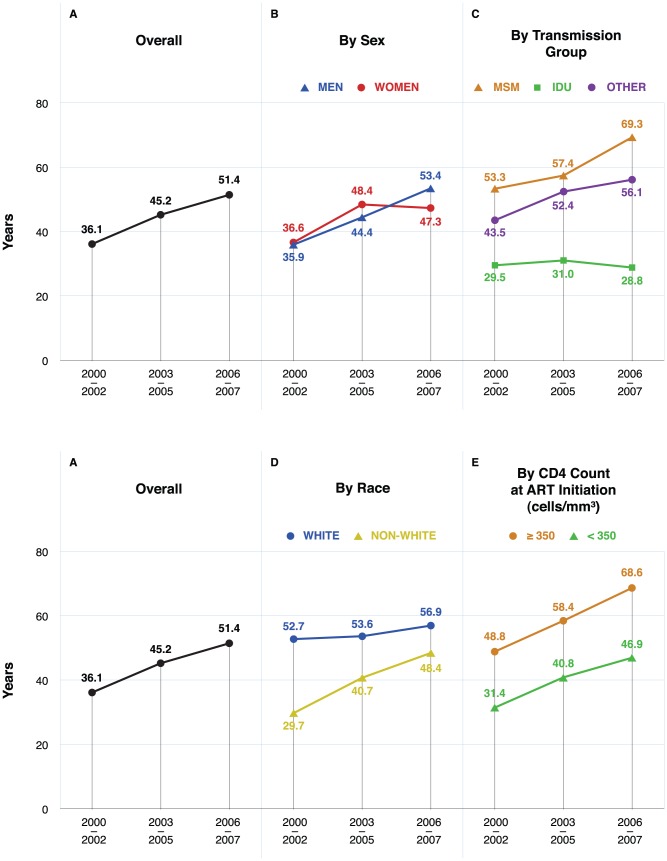
Mid-point life expectancy estimates at age 20 years in three calendar periods, overall and by sociodemographic characteristics, 2000–2007. Panel A: Life expectancy at age 20 years, overall. Panel B: Life expectancy at age 20 years, by sex. Panel C: Life expectancy at age 20 years, by transmission group. Panel D: Life expectancy at age 20 years, by race. Panel E: Life expectancy at age 20 years, by CD4 cell count (cells/mm^3^) at ART initiation.

**Table 4** and **Figure 2** show life expectancy at age 20 years by calendar period and by sociodemographic characteristics based on unweighted mortality rates. Life expectancy at age 20 years increased with calendar time, from 36.1 (SE 0.5) years in 2000–2002 to 51.4 (SE 0.5) years in 2006–2007. There was an increase in life expectancy among all groups over calendar time with the exception of individuals with a history of IDU, who had the lowest recorded life expectancies in all periods.

**Table 4:** Life expectancy estimates (LEE) and standard errors (SE) at age 20 years using unweighted mortality rates, by calendar period, 2000 to 2007.

	2000–2002 (LEE, SE)	2003–2005 (LEE, SE)	2006–2007 (LEE, SE)
**Overall**	36.1 (0.5)	45.2 (0.4)	51.4 (0.5)
**Sex**			
Men	35.9 (0.5)	44.3 (0.5)	53.4 (0.6)
Women	36.6 (1.0)	48.4 (0.8)	47.3 (0.5)
**Mode of transmission**			
Injection drug use	29.5 (0.9)	31.0 (0.7)	28.8 (0.4)
Men who have sex with men	53.3 (0.9)	57.4 (0.8)	69.3 (1.0)
Other transmission groups	43.5 (0.9)	52.4 (0.7)	56.1 (0.9)
**Race**			
White	52.7 (0.8)	53.6 (0.7)	56.9 (0.9)
Non-white	29.7 (0.6)	40.7 (0.5)	48.4 (0.6)
**CD4 cell count at start of ART (cells/mm^3^)**			
<350	31.4 (0.6)	40.8 (0.5)	46.9 (0.6)
≥350	48.8 (0.9)	58.4 (0.8)	68.6 (1.2)

Note: LEE, *life expectancy estimate (years)*; SE, *standard error*; ART, *combination antiretroviral therapy*.

## Discussion

Based on current patterns of ART use among participants observed from 2000 to 2007 in the NA-ACCORD, a 20-year-old individual on ART today in the U.S. or Canada would expect to live into their early 70 s, a life expectancy that approaches that of a 20-year-old person in the general population [12]. Life expectancy estimates for the general population at age 20 years in 2009 were 59.7 and 57.0 years for men and 63.9 and 61.7 years for women, in Canada and the U.S., respectively [12]. Indeed, given that many individuals living with HIV have demographic, clinical, and behavioral characteristics associated with greater morbidity and mortality than the general population [20], [21], the gap in life expectancy may be attributable to other lifestyle factors and not just HIV infection.

Importantly, our results are not confounded by previous antiretroviral use, as all participants were treatment-naive before initiating combination therapy. Weighted and unweighted rates overall and by calendar period produced similar results suggesting that our adjusted estimates are robust to any potential for informative censoring bias from the variables that were included in our model. Life expectancy does differ markedly by transmission group, race, and CD4 cell count at ART initiation; in particular, it is notably lower in individuals with a history of IDU, who are non-white, and who began ART with lower CD4 cell counts.

In the early years of ART availability, mortality was highest in the first year after ART initiation, but this has decreased over time. The modern ART era, in which mortality has become increasingly dependent on duration of ART, is characterized by a greater proportion of patients with longer follow-up. Therefore, the increase in life expectancy in the more recent time periods may reflect both a lower mortality rate in patients initiating ART and the mortality in those with a longer duration of ART use.

Our results are consistent with previous studies that have examined the life expectancy of HIV-positive individuals on ART [22]. While prior studies have observed similar decreases in mortality and increases in life expectancy among HIV-positive individuals as a result of ART [23], such findings have often been localized at the provincial or state level, and may now be less relevant to the current context of antiretroviral care [24], [25]. Furthermore, this is the first study to examine life expectancy in such a large sample of heterogeneous HIV-positive individuals on ART across the U.S. and Canada, and therefore provides novel data from this region.

The absence of significant differences in life expectancy by sex, and the higher life expectancy of men in the latest period suggests that there is in fact a sex differential in life expectancy in our study. In general, in high-income countries such as the U.S. and Canada we expect women to have higher life expectancies than men, which was not evident in our findings [12]. Similar life expectancies by sex or higher life expectancy for men suggest that women may be accessing HIV-related care at later stages of HIV disease than men in Canada and the U.S., which has significant public health ramifications. The sex differential, noted here, may also be explained in part due to the fact that general population comparisons better represent all men and women, while HIV-positive men here are weighted towards MSM and women toward IDU. Variation in levels of education and income, as well as access to the health care system, social stigma, and marginalization are other factors that may influence the observed differences by sex [26].

There are also considerable differences in life expectancy by HIV transmission group, with lower life expectancies reported in all periods for individuals with a history of IDU. This finding is consistent with previous findings from the ART Cohort Collaboration (a multisite cohort collaboration that includes seven sites in North America) and work done in British Columbia, Canada [2], [27]. We hypothesize a number of possible reasons for these differences, including increased comorbidity with greater non-AIDS related mortality, as well as challenges with ART adherence, active drug use, hepatitis C co-infection, housing instability, and lower socioeconomic status. Our findings here clearly illustrate that individuals with IDU history have not seen the increases in life expectancy that are evident in other groups.

Differences in life expectancy by race were also evident, with white individuals having higher life expectancies in all periods. As with persons with a history of IDU, these differences in life expectancy may be reflective of underlying differences in socioeconomic conditions, access to care, and health insurance coverage, suggesting an urgent need for strategies and programs to combat these inequities [28], [29]. However, we note that the gap in life expectancy between white and non-white individuals has decreased substantially, from 23.0 years in 2000–02 to 8.5 years in 2006–07.

Additional differences in life expectancies were noted by CD4 cell count at ART initiation in all periods. These findings may lend additional support to the earlier initiation of ART, in accordance with both the DHHS guidelines recommending universal therapy [30] and the 2012 International AIDS Society-USA (IAS-USA) guidelines, which recommend use of ART for all HIV-positive individuals regardless of CD4 cell count, except in the case of long-term non-progressors and elite controllers [3]. While our sample size limited our ability to analyze differences in life expectancy by more than two CD4 cell count strata, further analyses examining life expectancies for individuals starting ART with CD4 counts >500/mm^3^ would be beneficial.

In reviewing these findings, there are several potential limitations that readers of our work should consider. A caveat of life expectancy analyses in general is that they may underestimate more recent improvements in extending life since age-specific mortality rates are based on a given point in time and assumed to apply for the duration of an individual's life [22]. Also, our results can only be generalized to those newly initiating ART, and not those with previous exposure to antiretroviral therapy. Although this collaboration includes the largest number of HIV-positive individuals and broadest geographic distribution in the U.S and Canada analyzed to-date, these individuals may not be fully representative of the epidemic in the entire region. However, previous analyses of the NA-ACCORD indicate that it does represent the demography of the epidemic in the U.S. [31]. Next, cohorts in this collaboration may under-represent those at greatest risk of death, as such individuals are less likely to seek care or to remain under care at one clinic. Thus, cohorts that did not link to vital statistics may miss deaths that occurred outside of the clinic setting. However, most participants (88%) were from cohorts that did link to vital statistics data. To further investigate this issue, we conducted sensitivity analyses taking into account loss to follow-up, and were reassured to find little differences in our estimates. Variations due to small numbers may also affect our estimates, especially due to the truncation of age intervals at older ages and the small number of deaths observed for some time intervals. We addressed the impact of small numbers by smoothing rates and examining the distribution of deaths over a calendar period. Finally, due to the increased risk of age-related co-morbidities among HIV-positive adults, it is possible life expectancy may plateau or decrease in the future; it will be important to monitor life expectancy estimates as more adults age with HIV.

In conclusion, the results of this study document increasing longevity for individuals living with HIV in the U.S. and Canada. The marked increase in life expectancy at age 20 from 36.1 years in 2000–2002 to 51.4 years in 2006–2007 is a testament to the improvements and overall success of ART. However, large differences in life expectancy persist between certain sub-groups of patients. Future work should consider specific reasons for these life expectancy gains, overall and within each sub-group. These data will be vital to target priorities for improvements in health services. Finally, in addition to quantity, future work should consider quality of life, as the proportion of individuals aging with HIV continues to grow.

## Supporting Information

Appendix S1 **NA-ACCORD cohorts.**
(DOCX)

Appendix S2 **Using inverse probability weights to address bias due to loss to follow-up.**
(DOCX)

Appendix S3 **Sensitivity analyses: Restricting to cohorts with death from registries.**
(DOCX)

Appendix S4 **Sensitivity analyses: Restricting to adults initiating ART from 2000–2007.**
(DOCX)

## References

[1] Boyd MA (2009) Improvements in antiretroviral therapy outcomes over calendar time. Curr Opin HIV AIDS 4: 194–199. 19532050 10.1097/COH.0b013e328329fc8d

[2] ART Cohort Collaboration 2008 Life expectancy of individuals on combination antiretroviral therapy in high-income countries: a collaborative analysis of 14 cohort studies. Lancet 372: 293. 18657708 10.1016/S0140-6736(08)61113-7 PMC3130543

[3] Thompson MA, Aberg JA, Hoy JF, Telenti A, Benson C, et al (2012) Antiretroviral Treatment of Adult HIV Infection: 2012 Recommendations of the International Antiviral Society–USA Panel. JAMA 308: 387–402. 22820792 10.1001/jama.2012.7961

[4] Wood E, Kerr T, Marshall BDL, Li K, Zhang R, et al (2009) Longitudinal community plasma HIV-1 RNA concentrations and incidence of HIV-1 among injecting drug users: prospective cohort study. BMJ 338: b1649. 19406887 10.1136/bmj.b1649 PMC2675696

[5] Montaner JSG, Lima VD, Barrios R, Yip B, Wood E, et al (2010) Association of highly active antiretroviral therapy coverage, population viral load, and yearly new HIV diagnoses in British Columbia, Canada: a population-based study. Lancet 376: 532–539. 20638713 10.1016/S0140-6736(10)60936-1 PMC2996043

[6] Cohen MS, Chen YQ, McCauley M, Gamble T, Hosseinipour MC, et al (2011) Prevention of HIV-1 infection with early antiretroviral therapy. N Engl J Med 365: 493–505. 21767103 10.1056/NEJMoa1105243 PMC3200068

[7] Justice AC (2010) HIV and aging: time for a new paradigm. Curr HIV/AIDS Rep 7: 69–76. 20425560 10.1007/s11904-010-0041-9

[8] Deeks SG (2011) HIV infection, inflammation, immunosenescence, and aging. Annu Rev Med 62: 141–155. 21090961 10.1146/annurev-med-042909-093756 PMC3759035

[9] Mills EJ, Bakanda C, Birungi J, Chan K, Ford N, et al (2011) Life expectancy of persons receiving combination antiretroviral therapy in low-income countries: a cohort analysis from Uganda. Ann Intern Med 155: 209–216. 21768555 10.7326/0003-4819-155-4-201108160-00358

[10] Losina E, Schackman BR, Sadownik SN, Gebo KA, Walensky RP, et al (2009) Racial and sex disparities in life expectancy losses among HIV-infected persons in the United States: impact of risk behavior, late initiation, and early discontinuation of antiretroviral therapy. Clin Infect Dis 49: 1570–1578. 19845472 10.1086/644772 PMC2783631

[11] Palella FJ Jr, Baker RK, Buchacz K, Chmiel JS, Tedaldi EM, et al (2011) Increased mortality among publicly insured participants in the HIV Outpatient Study despite HAART treatment. AIDS 25: 1865–1876. 21811144 10.1097/QAD.0b013e32834b3537

[12] World Health Organization (2012) Life tables for WHO Member States. Available: http://www.who.int/healthinfo/statistics/mortality_life_tables/en/. Accessed 18 December 2012.

[13] Gange SJ, Kitahata MM, Saag MS, Bangsberg DR, Bosch RJ, et al (2007) Cohort profile: the North American AIDS Cohort Collaboration on Research and Design (NA-ACCORD). Int J Epidemiol 36: 294–301. 17213214 10.1093/ije/dyl286 PMC2820873

[14] Lima VD, Harrigan R, Bangsberg DR, Hogg RS, Gross R, et al (2009) The combined effect of modern highly active antiretroviral therapy regimens and adherence on mortality over time. J Acquir Immune Defic Syndr 50: 529–536. 19223785 10.1097/QAI.0b013e31819675e9 PMC3606956

[15] Chiang CL (1968) The life table and its construction. Introduction to Stochastic Processes in Biostatistics. New York: John Wiley and Sons. pp. 189–214.

[16] Bor J, Herbst AJ, Newell ML, Bärnighausen T (2013) Increases in adult life expectancy in rural South Africa: valuing the scale-up of HIV treatment. Science 339: 961–5. 23430655 10.1126/science.1230413 PMC3860268

[17] World Health Organization (2013) Life Expectancy. Available: www.who.int/topics/life_expectancy/en/. Accessed: 30 September 2013.

[18] Miniño AM, Murphy SL, Xu J, Kochanek KD (2011) Deaths: Final Data for 2008. National Vital Statistics Reports 59. 22808755

[19] Robins JM, Finkelstein DM (2004) Correcting for noncompliance and dependent censoring in an AIDS clinical trial with inverse probability of censoring weighted (IPCW) log-rank tests. Biometrics 56: 779–788. 10.1111/j.0006-341x.2000.00779.x 10985216

[20] Patel P, Hanson DL, Sullivan PS, Novak RM, Moorman AC, et al (2008) Incidence of types of cancer among HIV-infected persons compared with the general population in the United States, 1992–2003. Ann Intern Med 148: 728–736. 18490686 10.7326/0003-4819-148-10-200805200-00005

[21] Marmot M (2005) Social determinants of health inequalities. Lancet 365: 1099–1104. 15781105 10.1016/S0140-6736(05)71146-6

[22] Nakagawa F, May M, Phillips A (2013) Life expectancy living with HIV: recent estimates and future implications. Curr Opin Infect Dis 26: 17–25. 23221765 10.1097/QCO.0b013e32835ba6b1

[23] Wada N, Jacobson LP, Cohen M, French A, Phair J, et al (2013) Cause-specific life expectancies after 35 years of age for human immunodeficiency syndrome-infected and human immunodeficiency syndrome-negative individuals followed simultaneously in long-term cohort studies, 1984–2008. Am J Epidemiol 177: 116–25. 23287403 10.1093/aje/kws321 PMC3590031

[24] King JT, Justice AC, Roberts MS, Chang CCH, Fusco JS (2003) Long-term HIV/AIDS survival estimation in the highly active antiretroviral therapy era. Med Decis Making 23: 9–20. 12583451 10.1177/0272989X02239652

[25] Lima VD, Hogg RS, Harrigan PR, Moore D, Yip B, et al (2007) Continued improvement in survival among HIV-infected individuals with newer forms of highly active antiretroviral therapy. AIDS 21: 685–692. 17413689 10.1097/QAD.0b013e32802ef30c

[26] Simard EP, Fransua M, Naishadham D, Jemal A (2012) The influence of sex, race/ethnicity, and educational attainment on human immunodeficiency virus death rates among adults, 1993–2007. Arch Intern Med 172: 1591–8. 23045164 10.1001/archinternmed.2012.4508

[27] Lloyd-Smith E, Brodkin E, Wood E, Kerr T, Tyndall MW, et al (2006) Impact of HAART and injection drug use on life expectancy of two HIV-positive cohorts in British Columbia. AIDS 20: 445–450. 16439879 10.1097/01.aids.0000206508.32030.92

[28] Wolfe D, Carrieri MP, Shepard D (2010) Treatment and care for injecting drug users with HIV infection: a review of barriers and ways forward. Lancet 376: 355–366. 20650513 10.1016/S0140-6736(10)60832-X

[29] Rubin MS, Colen CG, Link BG (2010) Examination of inequalities in HIV/AIDS mortality in the United States from a fundamental cause perspective. Am J Public Health 100: 1053–1059. 20403885 10.2105/AJPH.2009.170241 PMC2866621

[30] DHHS: Panel on Antiretroviral Guidelines for Adults and Adolescents (2012) Guidelines for the use of antiretroviral agents in HIV-1-infected adults and adolescents. Available: aidsinfo.nih.gov/contentfiles/lvguidelines/. Accessed: 17 October 2012.

[31] Althoff KN, Buchacz K, Hall HI, Zhang J, Hanna DB, et al (2012) US trends in antiretroviral therapy use, HIV RNA plasma viral loads, and CD4 T-lymphocyte cell counts among HIV-infected persons, 2000 to 2008. Ann Intern Med 157: 325–335. 22944874 10.7326/0003-4819-157-5-201209040-00005 PMC3534765

