# Supplementary material for: Closing the Gap: Increases in Life Expectancy among Treated HIV-Positive Individuals in the United States and Canada
Source: PLoS One. 2013 Dec 18;8(12):e81355. doi: 10.1371/journal.pone.0081355 (PMC3867319; doi:10.1371/journal.pone.0081355)
Supplement: Appendix S1 — NA-ACCORD cohorts. (DOCX) [file pone.0081355.s001.docx]

**APPENDIX**

**S1. NA-ACCORD cohorts**

All cohorts participating in the NA-ACCORD (and their principal investigators) are listed below. Cohorts contributing participants to our life expectancy estimates are in italics.

*AIDS Link to the IntraVenous Experience (Gregory D. Kirk);*

*Adult AIDS Clinical Trials Group Longitudinal Linked Randomized Trials (Constance A. Benson, Ronald J. Bosch, and Ann C. Collier);*

Fenway Health HIV Cohort (Stephen Boswell, Chris Grasso, Ken Mayer)

*HAART Observational Medical Evaluation and Research (Robert S. Hogg, Richard Harrigan, Julio Montaner, Hasina Samji, and Angela Cescon);*

*HIV Outpatient Study (John T. Brooks and Kate Buchacz);*

*HIV Research Network (Kelly A. Gebo and Richard Moore);*

*Johns Hopkins HIV Clinical Cohort (Richard D. Moore);*

*John T. Carey Special Immunology Unit Patient Care and Research Database, Case Western Reserve University (Benigno Rodriguez);*

Kaiser Permanente Mid-Atlantic States (Michael A. Horbert);

*Kaiser Permanente Northern California (Michael A. Horberg, and Michael J. Silverberg);*

Longitudinal Study of Ocular complications of AIDS (Jennifer E. Thorne);

*Multicenter Hemophilia Cohort Study–II (James J. Goedert);*

*Multicenter AIDS Cohort Study (Lisa P. Jacobson);*

*Montreal Chest Institute Immunodeficiency Service Cohort (Marina B. Klein);*

Ontario HIV Treatment Network Cohort Study (Sean B. Rourke, Ann Burchell, and Anita R. Rachlis);

Retrovirus Research Center, Puerto Rico (Robert F. Hunter-Mellado and Angel Mayor)

*Southern Alberta Clinic Cohort (M. John Gill);*

*Studies of the Consequences of the Protease Inhibitor Era (Steven G. Deeks and Jeffery N. Martin);*

*University of Alabama at Birmingham 1917 Clinic Cohort (Michael S. Saag, Michael Mugavero, and James Willig);*

*University of North Carolina, Chapel Hill HIV Clinic Cohort (Joseph J. Eron, and Sonia Napravnik);*

*University of Washington HIV Cohort (Mari M. Kitahata and Heidi M. Crane);*

Veterans Aging Cohort Study (Amy C. Justice, Robert Dubrow, and David Fiellin);

*Vanderbilt-Meharry CFAR Cohort (Timothy R. Sterling, David Haas and Sam Stinnette);*

*Women’s Interagency HIV Study (Stephen J. Gange and Kathryn Anastos).*

**NA-ACCORD Executive Committee**:

Richard D. Moore, Michael S. Saag, Stephen J.Gange, Mari M. Kitahata, Rosemary G. McKaig, Amy C. Justice and Aimee M. Freeman.

**Epidemiology/Biostatistics Core:**

Stephen J. Gange, Alison G. Abraham, Bryan Lau, Keri N. Althoff, Jinbing Zhang, Jerry Jing, Elizabeth Golub, Shari Modur, David Hanna, Peter Rebeiro, Adell Mendes, and Cherise Wong.

**Data Management Core**:

Mari M. Kitahata, Stephen E. Van Rompaey, Heidi M. Crane, Eric Webster, Liz Morton, and Brenda Simon.

**Ethical Approval**:

The human subjects activities of the NA-ACCORD has been approved by the Johns Hopkins School of Medicine institutional review board (NA_00002683) as well as the local institutional review boards at each of the participating cohorts, as follows: Johns Hopkins Bloomberg School of Public Health Office for Research Subjects (ALIVE, MACS, WIHS), Harvard School of Public Health Human Subjects Administration (ALLRT), Providence Health Care Research Institute Office of Research Services (HOMER), Center for Disease Control Human Research Protection Office (HOPS), Johns Hopkins School of Medicine Office of Human Subjects Research (HIVRN, JHHCC), University Hospitals of Cleveland Institutional Review Board for Human Investigations (CWRU), Kaiser Foundation Research Institute Institutional Review Board (KPNC), National Cancer Institute Central Institutional Review Board (MHCS-II), McGill University Health Center Biomedical Research Ethics Board (MONT), University of Calgary Office of Medical Bioethics (SAC), University of California, San Francisco Office of Research (SCOPE), University of Alabama at Birmingham Institutional Review Board for Human Use (UAB), University of North Carolina at Chapel Hill Office of Human Research Ethics (UCHCC), University of Washington Office of Sponsored Programs, Human Subjects Division (UW), Vanderbilt University Institutional Review Board (VAND). All local cohorts have obtained written consent except the following: HOMER (Our IRB approves the retrospective use of anonymous administrative data without requiring consent. We provide an information sheet for participants in lieu of a consent form); KPNC (Our IRB provided a waiver of informed consent); MONT (Our IRB approves the anonymous use of data retrospectively abstracted from clinical care databases without requiring consent. Our patients sign a general waiver on opening a medical chart at the hospital but no specific study related consent); SAC (Written consent to treatment by all patients and use of anonymous administrative data approved by ethics); SCOPE (We were granted a waiver for informed consent by our IRB.); VAND (Patients included in our NA-ACCORD dataset do not provide informed consent. We use previously collected (“on the shelf”) clinical data. We do have IRB approval for NA-ACCORD studies). The need for informed consent for NA-ACCORD, as a study, is waived because IRB review determined that our research does not involve human subjects’ research under the DHHS or FDA regulations.
