# Supplementary material for: Closing the Gap: Increases in Life Expectancy among Treated HIV-Positive Individuals in the United States and Canada
Source: PLoS One. 2013 Dec 18;8(12):e81355. doi: 10.1371/journal.pone.0081355 (PMC3867319; doi:10.1371/journal.pone.0081355)
Supplement: Appendix S2 — Using inverse probability weights to address bias due to loss to follow-up. (DOCX) [file pone.0081355.s002.docx]

**S2. Using inverse probability weights to address bias due to loss to follow-up**

Addressing issues of loss to follow-up in observational studies can be complex. There may be an inherent bias in making inferences about individuals who are lost to follow-up compared to their uncensored counterparts. Fortunately, there are weighting methods that can correct for such biases, permitting valid comparisons. Inverse probability weighting (IPW) is an estimating tool from a class of techniques called marginal structural models that facilitate causal inference with observational data. In essence, IPW assigns each participant a weight equivalent to the inverse probability of remaining in the study at each interval based on the individual’s values of observed covariates and past exposures.

Our sample in this study consists of 22,937 treatment-naïve ART initiators participating in any one of the NA-ACCORD contributing cohorts. The study period was restricted to January 1, 2000 to December 31, 2007. We calculated crude mortality rates using participants’ person-time from the later of ART initiation, cohort inception date, or study start date, to the first of any of the following: death, loss to follow-up, or administrative censoring. We partitioned person-time into three distinct periods (Period 1: 1/1/2000-12/31/2002; Period 2: 1/1/2002-12/31/2005; Period 3: 1/1/2006-12/31/2007) to estimate period-specific mortality rates. From these mortality rates, life expectancy at age 20 years was estimated.

An indicator variable for participants who were lost to follow-up (i.e., those without death recorded and not administratively censored on December 31, 2007) was used in a pooled logistic regression model to estimate the inverse probability of loss to follow-up censoring weights. Weights were calculated as the ratio of predicted probabilities from two logistic regression models: the probability obtained from a model with baseline covariates divided by the probability obtained from a second model with baseline covariates as well as time-varying, period-specific covariates. Thus, each period for which an individual has contributed time will have an associated weight to account for the censoring due to loss to follow-up. We truncated the weights at the 1^st^ and 99^th^ percentiles [1]. These weights were then applied to Poisson regression models to estimate mortality rates.

During the overall study period, 27% (6,196/22,937) of participants were lost to follow-up (Table S1). Participants who were aged 35-44 and predominantly male, Black, MSM, or who had a lower CD4 cell count at baseline were more likely to be lost to follow-up compared to their counterparts who remained under observation.

| **Table S1. Descriptive Statistics by LTFU Censor Status (N=22,937)** | | | | | | |
| --- | --- | --- | --- | --- | --- | --- |
| Variable | Total N=22,937 | LTFU N=6,196 | | No LTFU N=16,741 | | p-value |
|  |  | N | % | N | % |  |
| **Age groups** |  |  |  |  |  | <0.0001 |
| 20-34 | 5,800 | 1,732 | 28 | 4,068 | 24 |  |
| 35-44 | 9,622 | 2,774 | 45 | 6,848 | 41 |  |
| 45-54 | 5,692 | 1,326 | 21 | 4,366 | 26 |  |
| 55+ | 1,823 | 364 | 6 | 1,459 | 9 |  |
| **Sex** |  |  |  |  |  | 0.17 |
| Male | 17,585 | 4,789 | 77 | 12,796 | 76 |  |
| Female | 5,352 | 1,407 | 23 | 3,945 | 24 |  |
| **Race** |  |  |  |  |  | <0.0001 |
| White | 8,643 | 2,244 | 36 | 6,399 | 38 |  |
| Black | 7,863 | 2,425 | 39 | 5,438 | 33 |  |
| Hispanic | 2,920 | 964 | 16 | 1,956 | 12 |  |
| **CD4 count** |  |  |  |  |  | <0.001 |
| <350 | 16,615 | 4,309 | 70 | 12,306 | 74 |  |
| 350+ | 6,322 | 1,860 | 30 | 4,435 | 27 |  |
| **HIV transmission** |  |  |  |  |  | <0.01 |
| IDU | 4,684 | 1,148 | 19 | 3,536 | 21 |  |
| MSM | 9,682 | 2,673 | 43 | 7,009 | 42 |  |
| Hetero | 7,832 | 2,030 | 33 | 5,802 | 35 |  |

To incorporate adjustment for early patient dropout, we used a stabilized weight method. This approach involves a repeated measures analysis using generalized estimating equations. The outcome for this analysis is a flag variable denoting whether the patient remained in the study. As this is a repeated measures analysis, we constructed our dataset as a person-period dataset whereby participants in our study must have contributed person time to at least one of the three calendar periods included in the analysis; lost to follow-up could have occurred at any time after entry. An indicator variable denoted whether an individual remained under observation in each person-period (1=censored or lost to follow-up; 0=uncensored or still under observation). This indicator (Cij) was then used as the outcome in two separate logistic regression models. The first model, referred to as the numerator model, incorporates time-fixed baseline covariates:

$$logit P\left( C_{ij} | X_{i} \right)= \beta X_{i}$$

where *i* represents participant, *j* represents the study period (1,2,3), and
*X_i_* represents the matrix of baseline covariates

The numerator model refers to the probability that participant *i* were uncensored during period _j_ conditional on a set of time-fixed covariates. Model selection criteria included likelihood ratio tests. All main effects and interaction terms with a p<0.1 were included in the final models. Baseline covariates that were included in the numerator model were sex, race, HIV transmission risk, cohort, calendar year of ART initiation, baseline age, baseline CD4, baseline viral load, and all relevant interactions.

A second model includes both time-fixed and time-varying covariates, which shall be referred to as the denominator model. The denominator model refers to the probability of remaining uncensored conditional on a set of time-fixed and time-varying covariates:

$$logit P\left( C_{ij}=0 | X_{i}, Z_{i} \right)= \alpha X_{i}+\gamma Z_{ij}$$

where *X_i_* represents the same matrix of baseline covariates in the numerator model and *Z_ij_* represents the matrix of time-varying covariates

Time-varying covariates eligible for the denominator model were measured at the start of a study period (if an individual survived from one study period to the next) and included age, CD4 cell count and log_10_ HIV-1 RNA. Only period-specific age remained statistically significant at p<0.1 and was included in the final denominator model.

To produce overall weights for each observation (person-period) in this analysis, we obtained the ratio of weights for each individual *i* during period *j*:

W_ij_ = $\frac{P\left( C_{ij} | X_{i} \right)}{P\left( C_{ij} | X_{i}, Z_{i} \right)}$

Wij can be interpreted as a stabilized weight to account for regular censoring in each person-period. This weight is equivalent to the inverse probability of subject *i* remaining uncensored during period *j*.

Table S2 presents the descriptive statistics for the stabilized weights. The medians of the period-specific stabilized weights obtained from fitting the repeated measures model for lost to follow-up can be seen in Table S2. The 1^st^ and 99^th^ percentiles of the weights were truncated. As a point of comparison, the overall stabilized weights were obtained by fitting a separate logistic regression model where the observation time was not split into periods. The period-specific weights were subsequently incorporated into weighted Poisson regression models to estimate lost to follow-up weighted mortality rates.

| **Table S2. Summary statistics for inverse probability weights for lost to follow-up** | | | | | |
| --- | --- | --- | --- | --- | --- |
| **Time** | Median (IQR) | Mean | Deaths | LTFU | Total Individuals |
| Period 1 (2000.00-2002.99) | 1.0018 (0.9919-1.0018) | 1.0000 | 482 | 1,600 | 10,941 |
| Period 2 (2003.00-2005.99) | 1.0006 (0.9929-1.0006) | 1.0004 | 691 | 2,405 | 15,713 |
| Period 3 (2006.00-2007.99) | 0.9997 (0.9909-0.9997) | 0.9997 | 449 | 2,191 | 17,759 |
| Overall (2000.00-2007.99) | 1.0014 (0.9874-1.0014) | 1.0007 | 1,622 | 6,196 | 22,937 |

A key assumption of this method is that it can only account for informative censoring associated with time-updated variables that were measured and included in the weighting models as listed above. The method continues to assume there is, conditionally on the variables in the model, no informative censoring due to unmeasured variables. As noted by others [2], this assumption is difficult to check without collection of additional data.

References

[1] Cole SR, Hernán MA. Constructing inverse probability weights for marginal structural models. Am J Epidemiol, 2008; 168(6): 656-664.

[2] Geng EH, Glidden DV, Bangsberg DR, Bwana MB, Musinguzi N, et al. A causal framework for understanding the effect of losses to follow-up on epidemiologic analyses in clinic-based cohorts: the case of HIV-infected patients on antiretroviral therapy in Africa. Am J Epidemiol, 2012; 175(10): 1080-1087.
