# Supplementary material for: Closing the Gap: Increases in Life Expectancy among Treated HIV-Positive Individuals in the United States and Canada
Source: PLoS One. 2013 Dec 18;8(12):e81355. doi: 10.1371/journal.pone.0081355 (PMC3867319; doi:10.1371/journal.pone.0081355)
Supplement: Appendix S3 — Sensitivity analyses: Restricting to cohorts with death from registries. (DOCX) [file pone.0081355.s003.docx]

**S3. Sensitivity analyses: Restricting to cohorts with death from registries**

In sensitivity analyses, we calculated mortality rates in the 14 cohorts that obtained information on deaths from linking to vital statistic registries and compared them to the four cohorts that do not link. Below are the mortality rates estimated in the 14 cohorts that link compared to those that do not by overall age and for each of our three study periods.

**Figure S1. Mortality rates in cohorts that link to vital statistics registries and those that do not by period**
