# Supplementary material for: Closing the Gap: Increases in Life Expectancy among Treated HIV-Positive Individuals in the United States and Canada
Source: PLoS One. 2013 Dec 18;8(12):e81355. doi: 10.1371/journal.pone.0081355 (PMC3867319; doi:10.1371/journal.pone.0081355)
Supplement: Appendix S4 — Sensitivity analyses: Restricting to adults initiating ART from 2000–2007. (DOCX) [file pone.0081355.s004.docx]

**S4. Sensitivity analyses: Restricting to adults initiating ART from 2000-2007**

In sensitivity analyses, we calculated mortality rates restricting to those who were observed to initiate ART from 2000 to 2007. Below are the mortality rates among these individuals by age category. Overall life expectancy at age 20 was 45.1 (SE 0.3).

| **Table S3. Cases, person-years, and rates per 1000 person-years stratified by age** | | | |
| --- | --- | --- | --- |
| Age Category | Deaths | Person-years | Mortality Rate (95% CI) |
| 20-<35 | 182 | 14,910 | 12.2 (10.6-14.1) |
| 35-<45 | 444 | 23,615 | 18.8 (17.1-20.6) |
| 45-<55 | 303 | 13,247 | 22.9 (20.4-25.6) |
| 55+ | 128 | 4,097 | 31.2 (26.3-37.1) |
